# Identification of Genes to Differentiate Closely Related *Salmonella* Lineages

**DOI:** 10.1371/journal.pone.0055988

**Published:** 2013-02-18

**Authors:** Qing-Hua Zou, Ren-Qing Li, Ye-Jun Wang, Shu-Lin Liu

**Affiliations:** 1 Department of Microbiology, School of Basic Medical Sciences, Peking University Health Science Center, Beijing, China; 2 Institute of Immunology, Beijing Center for Disease Control and Prevention, Beijing, China; 3 Genomics Research Center (one of The State-Province Key Laboratories of Biomedicine-Pharmaceutics of China), Harbin Medical University, Harbin, China; 4 Department of Microbiology and Infectious Diseases, University of Calgary, Calgary, Alberta, Canada; University of Osnabrueck, Germany

## Abstract

**Background:**

*Salmonella* are important human and animal pathogens. Though highly related, the *Salmonella* lineages may be strictly adapted to different hosts or cause different diseases, from mild local illness like gastroenteritis to fatal systemic infections like typhoid. Therefore, rapid and accurate identification of *Salmonella* is essential for timely and correct diagnosis of *Salmonella* infections. The current identification methods such as 16S rRNA sequencing and multilocus sequence typing are expensive and time consuming. Additionally, these methods often do not have sufficient distinguishing resolution among the *Salmonella* lineages.

**Methodologies/Principal Findings:**

We compared 27 completely sequenced *Salmonella* genomes to identify possible genomic features that could be used for differentiation of individual lineages. We concatenated 2372 core genes in each of the 27 genomes and constructed a neighbor-joining tree. On the tree, strains of each serotype were clustered tightly together and different serotypes were unambiguously separated with clear genetic distances, demonstrating systematic genomic divergence among the *Salmonella* lineages. We made detailed comparisons among the 27 genomes and identified distinct sets of genomic differences, including nucleotide variations and genomic islands (GIs), among the *Salmonella* lineages. Two core genes STM4261 and *entF* together could unambiguously distinguish all *Salmonella* lineages compared in this study. Additionally, strains of a lineage have a common set of GIs and closely related lineages have similar sets of GIs.

**Conclusions:**

*Salmonella* lineages have accumulated distinct sets of mutations and laterally acquired DNA (e.g., GIs) in evolution. Two genes *entF* and STM4261 have diverged sufficiently among the *Salmonella* lineages to be used for their differentiation. Further investigation of the distinct sets of mutations and GIs will lead to novel insights into genomic evolution of *Salmonella* and greatly facilitate the elucidation of pathogeneses of *Salmonella* infections.

## Introduction

The bacterial genus *Salmonella* encompasses a large number of lineages that cause a variety of diseases in humans or animals. In the United States, for example, *Salmonella* infections result in 15,000 hospitalizations and more than 400 deaths annually [1]. To date, more than 2500 lineages (Each *Salmonella* lineage was treated as individual species up to the 1930s, as both species and serotypes by the 1980s, and as serovars of one or two *Salmonella* species since late 1980s; currently a large number of journals still treat *Salmonella* lineages as species. See details in [2]) have been documented in the *Salmonella* genus [3]. All *Salmonella* bacteria are closely related but the individual lineages may differ greatly in pathogenic features, such as in host range or in the nature of diseases caused. For example, *Salmonella enterica* subspecies *enterica* serovar Typhi (*S.* Typhi) and *S.* Paratyphi A are strictly adapted to humans and cause typhoid, a serious systemic infection with a high mortality rate [4], whereas *S.* Typhimurium is a broad host-range pathogen, causing systemic infection in mice but only gastroenteritis in humans [5]. Similarly, the fowl-specific lineage *S.* Gallinarum causes typhoid-like disease and its close relative *S.* Pullorum, also fowl-specific, causes dysentery [6]. Although identification methods, such as the Kauffman-White scheme for serotyping [7] and DNA analysis for multilocus sequence typing [8], have been developed to differentiate the highly related but pathogenically distinct *Salmonella* lineages, these methods are either not available to most clinical laboratories or tedious and expensive. Therefore, in-depth comparative analyses of the *Salmonella* genomes are necessary to reveal unique features on them for possible use in convenient and accurate identification of these bacteria.

Genomic comparisons among representative *Salmonella* pathogens, based on physical mapping [9], [10], provided the first evidence indicating that genomes of individual *Salmonella* lineages differ mainly in their specific sets of insertions [11], [12], [13], [14], [15], [16], [17], [18]. Rearrangements of the genomes have been characterized in host-adapted *Salmonella* pathogens such as *S.* Typhi [19], [20], [21] and *S.* Paratyphi C [22], but they are likely the consequence rather than the cause of genomic divergence, thus providing little if any help in the differentiation of individual *Salmonella* lineages. Nucleotide changes on the other hand, also as main events during *Salmonella* genomic evolution, have been evidenced as early as in the physical mapping era by the distinct endonuclease cleavage patterns among different *Salmonella* lineages [23], [24], but how much contribution nucleotide change may make to genomic divergence and whether certain nucleotide changes may be used as “signatures” of some *Salmonella* lineages for identification remain largely unexplored.

Genomic comparisons at the sequence level validated our previous findings with higher resolution, revealing distinct sets of independently accumulated nucleotide variations, the identity of exogenous DNA insertions and the precise endpoints of large scale genomic rearrangements [5], [25], [26], [27], [28], [29]. In this study, we analyzed genomic divergence among twenty seven sequenced *Salmonella* strains with a focus on the sequence variations of core genes. This comprehensive analysis provides new parameters for the differentiation of different *Salmonella* lineages and will significantly facilitate further studies towards the elucidation of the genetic basis of differential host ranges and distinct pathogenic properties of the *Salmonella* pathogens.

## Materials and Methods

### Genomic Sequences

The 27 complete genome sequences of *Salmonella* in the NCBI genome database as of March, 2012, were downloaded and used in this work (Table 1). These strains belong to 15 *Salmonella* lineages.

**Table 1:** Genomes analyzed in this study.

Accession No.	Lineage	Strain No.	Genome Size (bp)
AE006468	*S.* Typhimurium	LT2	4,857,432
AP011957	*S.* Typhimurium	T000240	4,954,814
FN424405	*S.* Typhimurium	D23580	4,879,400
CP002614	*S.* Typhimurium	UK-1	4,817,868
CP002487	*S.* Typhimurium	ST4/74	4,878,013
FQ312003	*S.* Typhimurium	SL1344	4,878,012
CP001363	*S.* Typhimurium	14028S	4,870,265
CP001120	*S.* Heidelberg	SL476	4,888,768
CP000886	*S.* Paratyphi B	SPB7	4,858,887
CP000857	*S.* Paratyphi C	RKS4594	4,833,080
AE017220	*S.* Choleraesuis	SC-B67	4,755,700
CM001062	*S.* Choleraesuis	SCSA50	4,740,379
CM001151	*S.* Dublin	SD3246	4,842,911
CP001144	*S.* Dublin	CT_02021853	4,8429,08
AM933172	*S.* Enteritidis	P125109	4,685,848
CP003047	*S.* Pollorum	RKS5078	4,637,962
AM933173	*S.* Gallinarum	287/91	4,658,697
CM001153	*S.* Gallinarum	SG9	4,658,698
CP001113	*S.* Newport	SL254	4,827,641
CP001127	*S.* Schwarzengrund	CVM19633	4,709,075
CP001138	*S.* Agona	SL483	4,798,660
AE014613	*S.* Typhi	Ty2	4,791,961
NC_003198	*S.* Typhi	CT18	4,809,037
CP000026	*S.* Paratyphi A	ATCC 9150	4,585,229
FM200053	*S.* Paratyphi A	AKU_12601	4,581,797
CP000880	*S. arizonae*	RSK2980	4,600,800
NC_015761	*S. bongori*	NCTC 12419	4,460,105

### Core Gene Variation Analysis among the *Salmonella* Strains

The coding sequences of the 27 genomes listed in Table 1 were downloaded and placed into one file. The coding sequence of *S.* Typhimurium LT2 was used as query sequence for core gene identification by using NCBI Basic Local Alignment Search Tool (BLAST), with the criteria being set at identity >75% and e-value <1e–10. Matches that do not conform to chromosomal co-linearity were removed manually. Multiple alignments for each core gene cluster from all strains were performed using Clustal W. Nucleotides on each position were compared among the 27 strains for nucleotides specific to individual lineages.

### Determination of Phylogenetic Relationships among the *Salmonella* Strains

All core gene sequences were aligned by Clustal W and concatenated for use in constructing a phylogenetic tree by the neighbor-joining method with the MEGA software (version 5.0). Evolutionary distances were estimated by the Maximum Composite Likelihood (MCL) method. The reliability of the neighbor-joining tree was estimated by bootstrap analysis with 1000 replicate data sets, and the bootstrap values supporting each cluster are shown at the nodes.

For a protein-based phylogenetic tree, we did BLAST searches of the deduced products of STM4261 and *entF* in *S.* Typhimurium LT2 against the NCBI non-redundant database. Homologous protein sequences were downloaded and aligned by ClustalW multiple alignment program in the Bioedit software, and a phylogentic tree was constructed by MEGA 5.

### GO Annotation

The gene information number of each core gene in LT2 was extracted and then mapped to Gene Ontology (GO) terms. The mapping script and the GO accession-GO term script were written by the authors.

### Comparison of Genomic Islands

Sequences of genomic islands were obtained by Island Viewer (http://www.pathogenomics.sfu.ca/islandviewer/query.php) and searched by BLAST against the 27 genomes. Sequences with coverage >90% and e-value <1e–10 were considered as positive results.

## Results

We compared 27 completely sequenced *Salmonella* genomes (of 15 lineages). The chromosome sizes range from 4,460,105 bp (*S. bongori*) to 4,954,814 bp (*S.* Typhimurium T000240). Interestingly, the two human-restricted *S.* Paratyphi A strains AKU_12601 and ATCC 9150 are among the isolates with the smallest chromosomes (4,581,797 bp and 4,585,229 bp, respectively), followed by the three fowl-adapted *S.* Pullorum and *S.* Gallinuarum strains (4,637,962 bp, 4,658,697 bp and 4,658,698 bp; Table 1). The two human-restricted typhoid agent *S. typhi* strains Ty2 and CT18 have intermediate chromosome sizes (4,791,961 bp and 4,809,037 bp, respectively), whereas the host-generalist *S.* Typhimurium strains have much larger chromosome sizes (4,817,868 to 4,954,814 bp; Table 1), suggesting a tendency towards smaller genome sizes of the host-adapted bacteria during host adaptation.

### Identification of Core Genes

We define core genes as the orthologs shared by all compared genomes. A total of 2372 core genes were found for the 27 strains, accounting for over a half of the coding sequences in each strain (Table S1). In order to determine the functional profiles of the core genes, we classified these genes based on Gene Ontology (GO) terms and found most of the terms are essential for living cells such as ATP binding, metal ion binding, DNA binding, transporter activity, etc.

**Figure 1:**
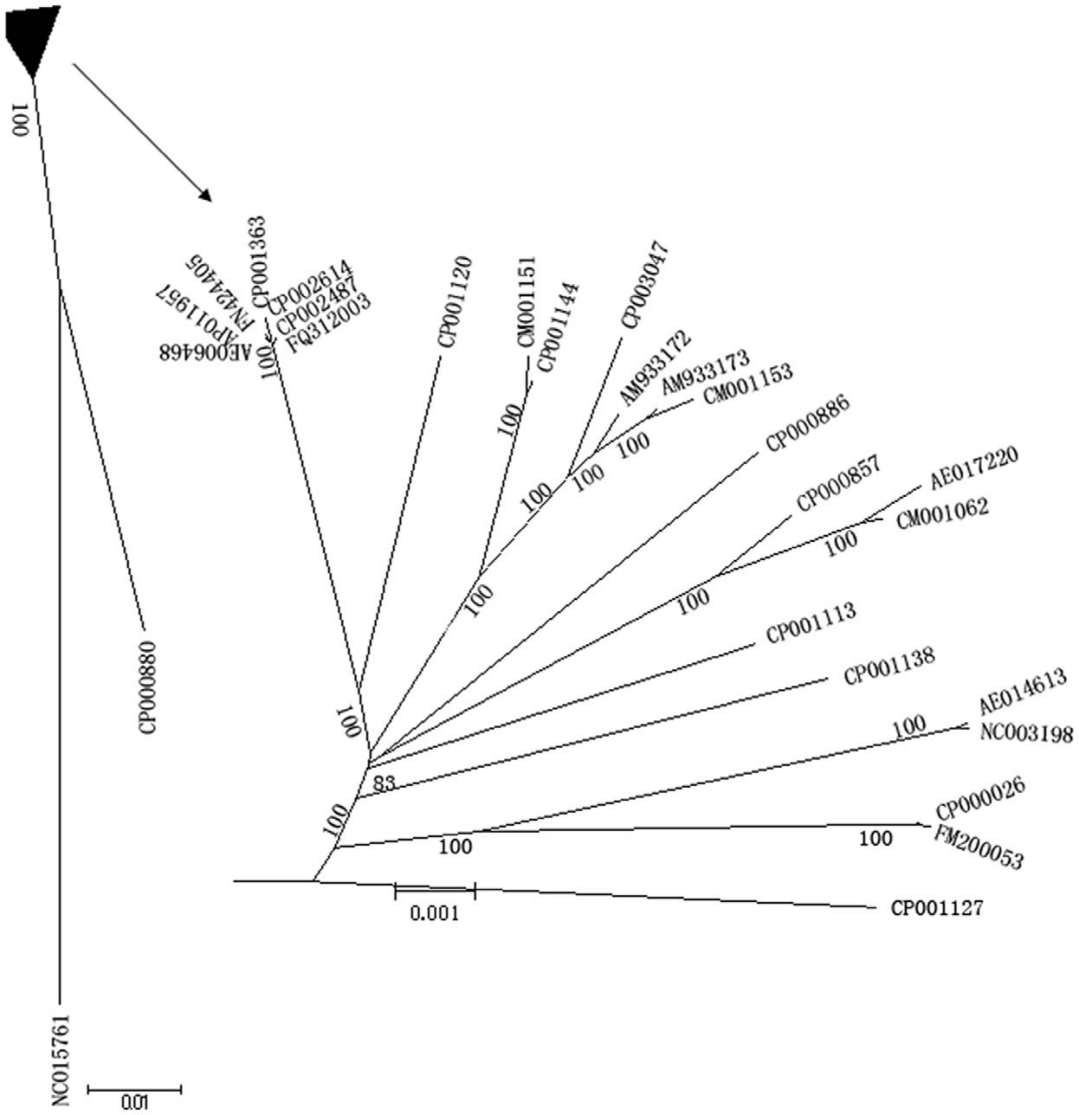
Phylogenetic trees of the 27 *Salmonella* strains based on the core genome. Accession numbers are used for the bacterial strains (See Table 1).

To reveal the evolutionary relationships among these strains, we built a phylogentic tree based on these core genes. As shown in Figure 1, *S. bongri* and *S. arizonae* lie at one end of the tree and all the other strains lie at the other end, with a strikingly long genetic distance between the two ends. The core genome tree shows similar lengths of the *S.* Typhi and *S.* Paratyphi A strains from other *Salmonella* lineages, indicating similar divergence times for the two human-restricted pathogens. Similar situations were seen between *S.* Choleraesuis and *S.* Paratyphi C, and among *S.* Gallinarum, *S.* Pullorum and *S.* Enteritidis. This observation is in agreement with previous findings that were based on analyses of shared genes (*S.* Paratyphi C and *S.* Choleraesuis [27]) and pseudogenes (*S.* Gallinarum and *S.* Enteritidis [30]).

### Genomic Variation in Core Genes among Different *Salmonella* Lineages

**Figure 2:**
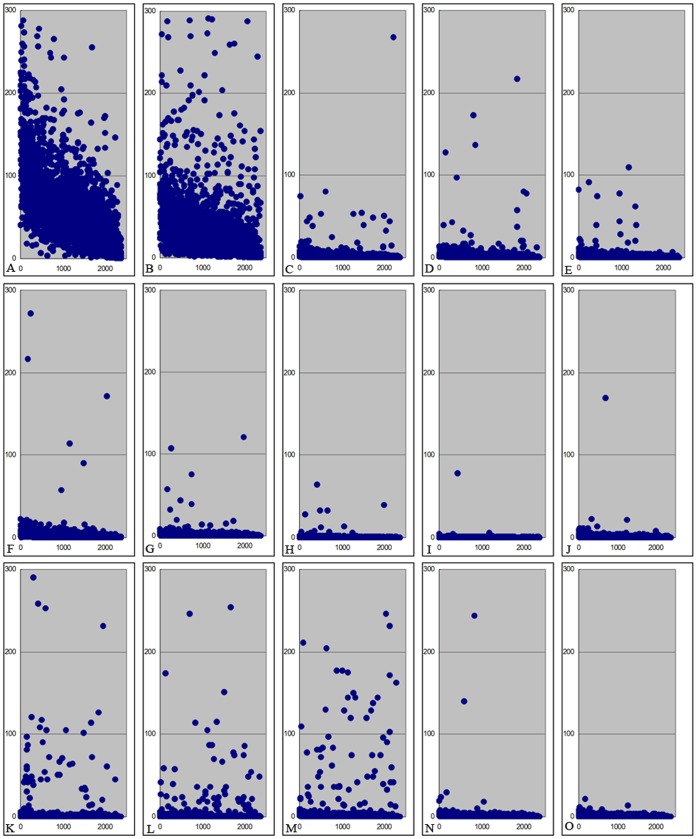
Numbers of lineage-specific nucleotides within the core gene sets. A, *S. bongori*; B, *S. arizonae*; C, *S.* Paratyphi A; D, *S.* Typhi; E, *S.* Agona; F, *S.* Schwarzengrund; G, *S.* Newport; H, *S.* Gallinarum; I, *S.* Enteritidis; J, *S.* Dublin; K, *S.* Choleraesuis; L, *S.* Paratyphi C; M, *S.* Paratyphi B; N, *S.* Heidelberg; O, *S.* Typhimurium.

As divergence levels of core genes reflect the evolutionary relationships among the bacteria, it is of great importance to determine the actual sequence variations in the genes. We extracted the 2372 core genes from each of the 27 genomes and aligned them for comparisons. We found that nucleotide variations are widely distributed in the core genes among the 15 lineages, with each lineage having a distinct set of nucleotide differences. We searched for lineage-specific nucleotides shared by strains of the same lineage and different from other lineages. Very interestingly, the 2372 core genes each have at least one nucleotide variation specific to a lineage, which may possibly be used as the signature of the corresponding lineage. Of special significance is the fact that the numbers of lineage-specific nucleotides on each core gene are different among different lineages, which may reflect distinct selection pressures on the core genome of different lineages when they were adapting to different niches. Figure 2 illustrates the numbers of specific nucleotides on each core gene in the 15 lineages. Four core genes, i.e., *cpsG, nuoG*, STM2397 and STM4261, have adequate resolution power to differentiate the 15 lineages, and another set of six core genes, *srfC, napA, yhgE, priA, cpdB* and *entF,* can distinguish 14 out of the 15 lineages.

### Phylogenetic Tree Based on the Deduced Amino Acid Sequences of STM4261 and *entF*

For the core genes that can distinguish most of the *Salmonella* lineages, the deduced amino acid sequences were compared to see whether the amino acids would also have similar discriminating resolution. When the deduced amino acids were compared, we found that some nucleotide variations are synonymous, thus losing some differentiation capability at the deduced protein level. However, two proteins, one being a putative inner membrane protein encoded by STM4261 and the other being enterobactin synthetase component F encoded by *entF*, can each distinguish 14 out of the 15 *Salmonella* lineages. Whereas the product of STM4261 is not specific for *S.* Gallinarum and EntF is not specific for *S.* Enteritidis, when combined, the two proteins can distinguish all 15 lineages.

STM4261 is located in the SPI-4 of *S.* Typhimurium. Previous studies demonstrate that when this gene was inactivated by transposon insertion in *S.* Typhimurium, the colonization potency of the bacteria to calf was decreased [31]. Previous studies show that *entF* is associated with the colonization of *S.* Typhimurium ST4/74. When inactivated by transposon insertion, the colonization of *S.* Typhimurium ST4/74 to calf and chicken became significantly decreased [31].

**Figure 3:**
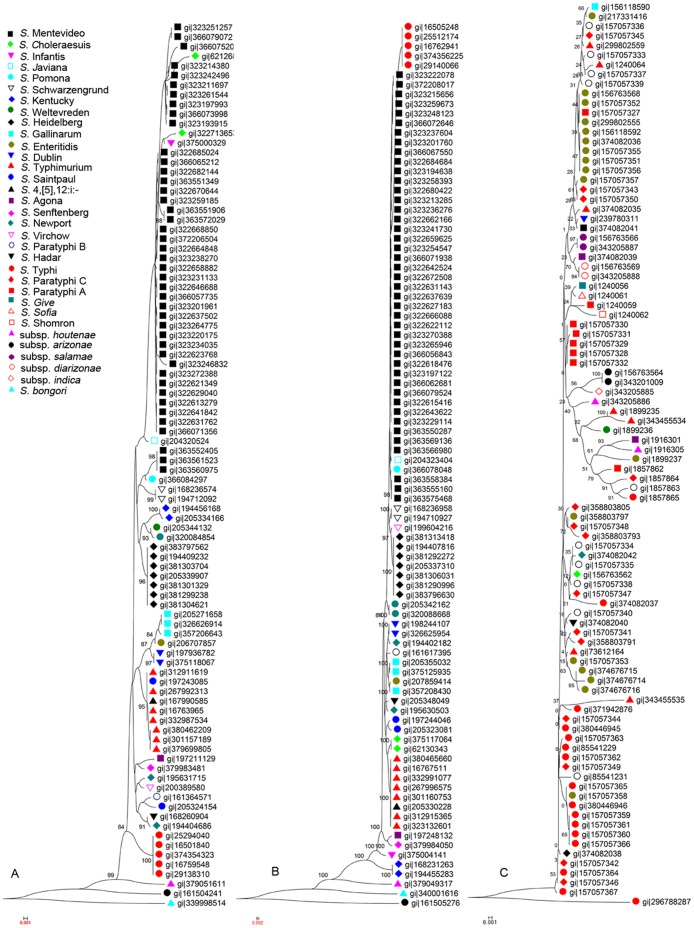
Phylogenetic trees, based on the deduced amino acid sequences of *entF* (A), STM4261 (B) and 16S rRNA (C).

Prompted by the excellent differentiation power of the two proteins for the 15 *Salmonella* lineages compared, we attempted to see whether they could distinguish additional *Salmonella* lineages. The deduced amino acid sequences of STM4261 and *entF* of *S.* Typhimurium LT2 were blasted against the NCBI non-redundant protein database. We found 92 *Salmonella* strains that have homologous proteins with the products of both STM4261 and *entF*, so these strains were selected for evaluation. The homologous protein sequences in the 92 strains were extracted and aligned by BioEdit, and then phylogenetic trees were built based on the two protein sequences. The 92 strains contain 43 *S.* Montevideo strains, which were isolated from a single outbreak [32]. Figure 3 shows a phylogenetic tree generated from EntF (Figure 3A) and one from STM4261 (Figure 3B). On the two trees, bacterial strains of the same lineage cluster tightly together, with minor exceptions. For example, on the EntF tree, the *S.* Montevideo strains were mixed with two *S.* Choleraesuis strains, one *S.* Infantis strain and one *S.* Javiana strain; additionally, the *S.* Typhimurium strains were mixed with *Salmonella* serotype 4, [5],12:i:- and *S.* Saintpaul. On the tree of the deduced STM4261 product, the *S.* Montevideo strains were mixed with *S.* Javiana and *S.* Pomona, and the *S.* Gallinarum strains were mixed with *S.* Enteritidis. However, when combined, the two deduced proteins could unambiguously distinguish strains of different *Salmonella* lineages, as shown by *S.* Choleraesuis, the two strains of which were separated on the EntF tree but clustered tightly together on STM4261 tree. Conversely, *S.* Gallinarum strains were not clustered closely on the STM4261 tree but were clustered well together on the EntF tree.

### Comparison of Phylogenetic Trees of STM4261 and EntF with those of 16S rRNA Sequences

The use of small subunit ribosomal RNA (16S rRNA in prokaryotes and 18S rRNA in eukaryotes) gene sequences for phylogenetic studies has revolutionized the natural classification of all life forms [33], [34], [35], [36]. Having demonstrated the excellent resolving power of STM4261 and *entF* for *Salmonella* lineages, we compared their performance with 16S rRNA. We searched the NCBI database for *Salmonella* 16S rRNA gene sequences and constructed a phylogentic tree for 16S rRNA gene sequences from 94 *Salmonella* strains (Figure 3C). Consistent with previous studies, 16S rRNA sequences did not have adequate resolution to differentiate the *Salmonella* lineages, further demonstrating the value of STM4261 and EntF in the identification of the *Salmonella* pathogens.

### Laterally Acquired Genomic Islands in the *Salmonella* Lineages – Contribution to Genomic Divergence and Potential Usage in the Bacterial Differentiation

**Figure 4:**
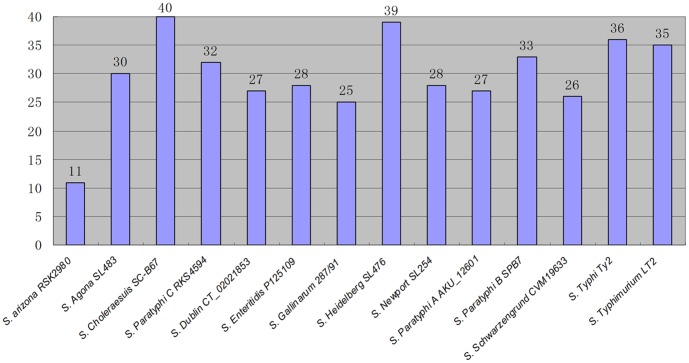
Numbers of GIs in representative strains of the sequenced *Salmonella* lineages.

Microbial genomes do not merely evolve through the slow accumulation of mutations, but also, and often more dramatically, by taking up new DNA in a process called horizontal gene transfer [25], [37], [38], [39], [40], [41]. The acquisition of new traits can take place not only via the incorporation of single genes, but also through the acquisition of large gene clusters, termed Genomic Islands (GIs). Even though many GIs have unknown functions, some of them have been demonstrated to play important roles in pathogenicity. Using Island Viewer, we analyzed the *Salmonella* genomes and obtained 417 GIs from 14 strains; the number of GIs in each strain is listed in Figure 4. Whereas some GIs distribute in more than one lineages, some are unique to a single lineage, such as the 104,005 bp GI, which lies only in the genome of *S.* Schwarzengrund CVM19633, with most of its genes encoding phage elements. Of great significance, strains of the same *Salmonella* lineage usually share a distinct set of GIs. As shown in Table S2, for example, most of the 35 GIs identified in *S.* Typhimurium LT2 were also present in other strains of *S.* Typhimurium, but only 12 to 22 are present in the strains of other lineages; similar situations were seen also in other lineages such as *S.* Paratyphi A and *S.* Typhi. Interestingly, closely related lineages tend to have a similar set of GIs. An example is the comparison of *S.* Choleraesuis and *S.* Paratyphi C, which are much more closely related to each other than either of them to any other lineages [27]: as many as 32 of the 40 GIs in the genome of *S.* Choleraesuis SC-B67 are present in the genome of *S.* Paratyphi C RKS4594, with only 10 to 21 being present in the strains of other lineages. Similarly, as many as 28 of the 32 GIs in *S.* Paratyphi C RKS4594 are present in the genome of *S.* Choleraesuis SC-B67. Other closely related lineages such as *S.* Enteritidis, *S.* Dublin and *S.* Gallinarum, also have similar sets of GIs (Table S2). This information may be used for evolutionary studies of the *Salmonella* lineages and provides further parameters for bacterial differentiation in a clinical setting.

## Discussion

In this study, we compared sequences of the core genes and GIs among the *Salmonella* strains that have the whole genome sequences available in NCBI (http://www.ncbi.nlm.nih.gov/) to look for genomic features that can be used to differentiate the *Salmonella* lineages. We identified distinct nucleotide variations common to strains of the same *Salmonella* lineage and different among the *Salmonella* lineages, including those in STM4261 and *entF*, the deduced amino acid sequences of which, when combined, could unambiguously distinguish all 15 *Salmonella* lineages compared in this study; the resolution power of these sequence variations were further validated in 92 additional *Salmonella* strains. These results reflect genetic isolation of the *Salmonella* lineages from one another during their evolution in distinct niches, such as different hosts (e.g., humans versus chickens) or different infection sites even in the same host (e.g., local versus systemic infections). Elucidation of the adaptation value of certain mutations accumulated in response to the environmental changes will lead to novel insights into the molecular basis of bacterial evolution.

As outbreaks of *Salmonella* infections are still serious problems threatening the human health, rapid and reliable sub-typing of epidemic strains is important for the identification of infectious agents in the outbreaks and the monitoring of trends. Currently, the most widely employed typing methods for bacteria include multilocus sequence typing (MLST) [42], pulsed field gel electrophoresis (PFGE) [43], sequencing of 16S rRNA genes, etc. However these methods are either time consuming (e.g., MLST, which requires the analysis of seven genes instead of two as with STM4261 and *entF*) or insufficient to clearly distinguish between closely related bacteria (e.g., 16S rRNA gene sequences, which are too conservative to have adequate resolution in differentiating the highly related *Salmonella* lineages). The uniqueness of the combined use of STM4261 and *entF* will provide convenient and accurate new methods for the identification and differentiation of *Salmonella* lineages for the purposes of clinical diagnosis.

Many *Salmonella* genomic islands are known to play important roles in virulence, with some being implicated in host specificity or invasiveness of the bacteria [44]. The GIs received by different *Salmonella* lineages vary in size and content and, usually, strains of the same lineage share a similar set of GIs. The fact that the GIs in bacteria of different lineages are very different suggests a major driving force of GIs for the evolution of these bacteria into different niches. Several studies support the convergent evolution model of the human-adapted typhoid agents [27], [28], [45], although genes directly contributing to the typhoid phenotypes remain to be identified. Since most of the GIs have a phage origin and many of their genes encode proteins responsible for fimbriae, O-antigen converstion, lipopolysaccharide biosynthesis and acetyltransferase, which will certainly influence the biological and, especially, pathogenic properties of *Salmonella*, this study reiterates the importance of bacteriophages in the evolution.

### Conclusions

*Salmonella* lineages have accumulated distinct sets of nucleotide mutations and laterally acquired DNA (e.g., GIs) in evolution. Two genes *entF* and STM4261 have diverged sufficiently among the *Salmonella* lineages to be used for their differentiation. Further investigation of the distinct sets of mutations and GIs will lead to novel insights into genomic evolution of *Salmonella* and greatly facilitate the elucidation of pathogeneses of *Salmonella* infections.

## Supporting Information

Table S1 **Core genes of the 27** ***Salmonella*** **strains.**
(XLS)

Table S2 **GIs in the genome of LT2 and its distribution in other strains.**
(XLS)
